# Predicted Relative Occupancy Probability of Raccoon Dogs (*Nyctereutes procyonoides*) in Northeast Beijing Using Bayesian Occupancy Models

**DOI:** 10.1002/ece3.73192

**Published:** 2026-03-14

**Authors:** Keisuke Miyamoto, Xiaofeng Luan

**Affiliations:** ^1^ School of Ecology and Nature Conservation Beijing Forestry University Beijing China

**Keywords:** anthropogenic factors, Beijing, nature reserve, occupancy model, raccoon dog, species distribution

## Abstract

Beijing has designated the raccoon dog ( *Nyctereutes procyonoides* ) as a protected species, and its protection status has been elevated to the national level in China. However, current knowledge of the species in Beijing is largely derived from localised studies, and region‐wide assessments remain lacking, despite its high conservation priority. In particular, understanding species distribution is fundamental to both ecology and conservation. This study evaluated raccoon dog occurrence patterns across environmental gradients and their spatial distribution in northeast Beijing, using Bayesian occupancy models based on camera‐trap detection–nondetection data. We examined the effects of environmental and anthropogenic factors; following model selection, minor road density and human population density were retained as key predictors. Predicted relative occupancy probability declined markedly with increasing densities of both variables, suggesting avoidance of human‐disturbed areas. Spatial predictions indicated higher predicted relative occupancy probabilities within nature reserves and in the northern part of the study area, while lower predicted occupancy in the southern part reflects the concentration of human‐related factors. These findings provide the first region‐wide assessment of raccoon dog occupancy in northeast Beijing, highlighting the importance of accounting for anthropogenic pressures. They also provide a regional baseline of raccoon dog distribution and offer insights to inform conservation planning and management.

## Graphical abstract

We assessed raccoon dog ( *Nyctereutes procyonoides* ) occupancy in northeast Beijing using camera‐trap data. Occupancy was higher in nature reserves and northern areas, but declined with increasing human population and minor road densities, highlighting the impact of anthropogenic pressures.

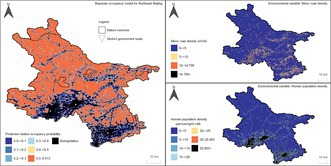

## 1 Introduction

In 2008, the raccoon dog was designated as a Class I protected species at the municipal level in Beijing. Since 2021, the status has been elevated to Class II under the List of National Key Protected Wild Animals. Now, the populations in South China are highly threatened (Lau et al. 2010). This designation reflects the conservation status of raccoon dogs in China, especially the unfavourable situation in Beijing. However, in Beijing, information on this species remains scarce and is limited mainly to local studies conducted in the Songshan National Nature Reserve (An et al. 2024; Fan et al. 2020) and Sizuolou Municipal Nature Reserve (Miyamoto et al. 2025). Given that raccoon dogs are threatened across China but not thoroughly researched, it is essential to address the existing knowledge gaps across the region.

Beijing, the capital of China, is located in northern China and consists of 18 districts. The northeastern districts are classified as part of the ecological conservation areas, which also correspond to regions with high forest vegetation (Mao et al. 2021). These areas exhibit a higher ecological and environmental quality index, reflecting superior ecological conditions (Beijing Municipal Ecology and Environment Bureau 2025). Within this environmental context, some areas are designated as nature reserves, which form the foundation of biodiversity conservation in China and play a crucial role in protecting biodiversity (Dayuan and Mingkang 1994; Wang et al. 2015; Zhang 2018). In particular, several of the largest municipal‐level nature reserves are concentrated in Huairou, Miyun and Pinggu districts (Beijing Municipal Bureau of Landscape and Forestry 2020), protecting forest vegetation and associated ecosystems (Dayuan and Mingkang 1994).

Understanding species distribution is fundamental to both ecology and conservation (Duscher and Nopp‐Mayr 2017; Fourcade et al. 2014; Richardson and Whittaker 2010). To this end, many Species Distribution Models (SDMs) have been developed to characterise species niches and assess habitat suitability (Richardson and Whittaker 2010). These models have proven effective in describing the natural distributions of species (Elith and Leathwick 2009). In the case of raccoon dogs, Diao et al. (2022) conducted the first study focusing on urban populations in China, using presence‐only data to demonstrate how SDM can support management planning in Shanghai. However, a review of current practice reveals that presence‐only data are used in more than half of correlative SDM studies (Guillera‐Arroita et al. 2015), although a key limitation of presence‐only data is that locations without detections cannot be distinguished between actual absence and imperfect detection (Ishihama 2017). Thus, despite numerous attempts, presence‐only data alone are inadequate for accurately estimating a species' true prevalence and spatial distribution (Fithian and Hastie 2013; Guillera‐Arroita et al. 2015). Occupancy models provide a flexible framework for incorporating both covariate information and imperfect detection (MacKenzie et al. 2002).

Given the limited knowledge of raccoon dogs in Beijing and growing conservation concerns, this study aimed to evaluate their occurrence patterns across environmental gradients and spatial distributions. Specifically, we addressed three objectives: (1) evaluating the performance of the Bayesian occupancy models, (2) interpreting the effects of environmental variables on predicted relative occupancy probability and (3) examining the spatial patterns of predicted occupancy and their ecological implications.

## 2 Methods

### 2.1 Study Area

**FIGURE 1:**
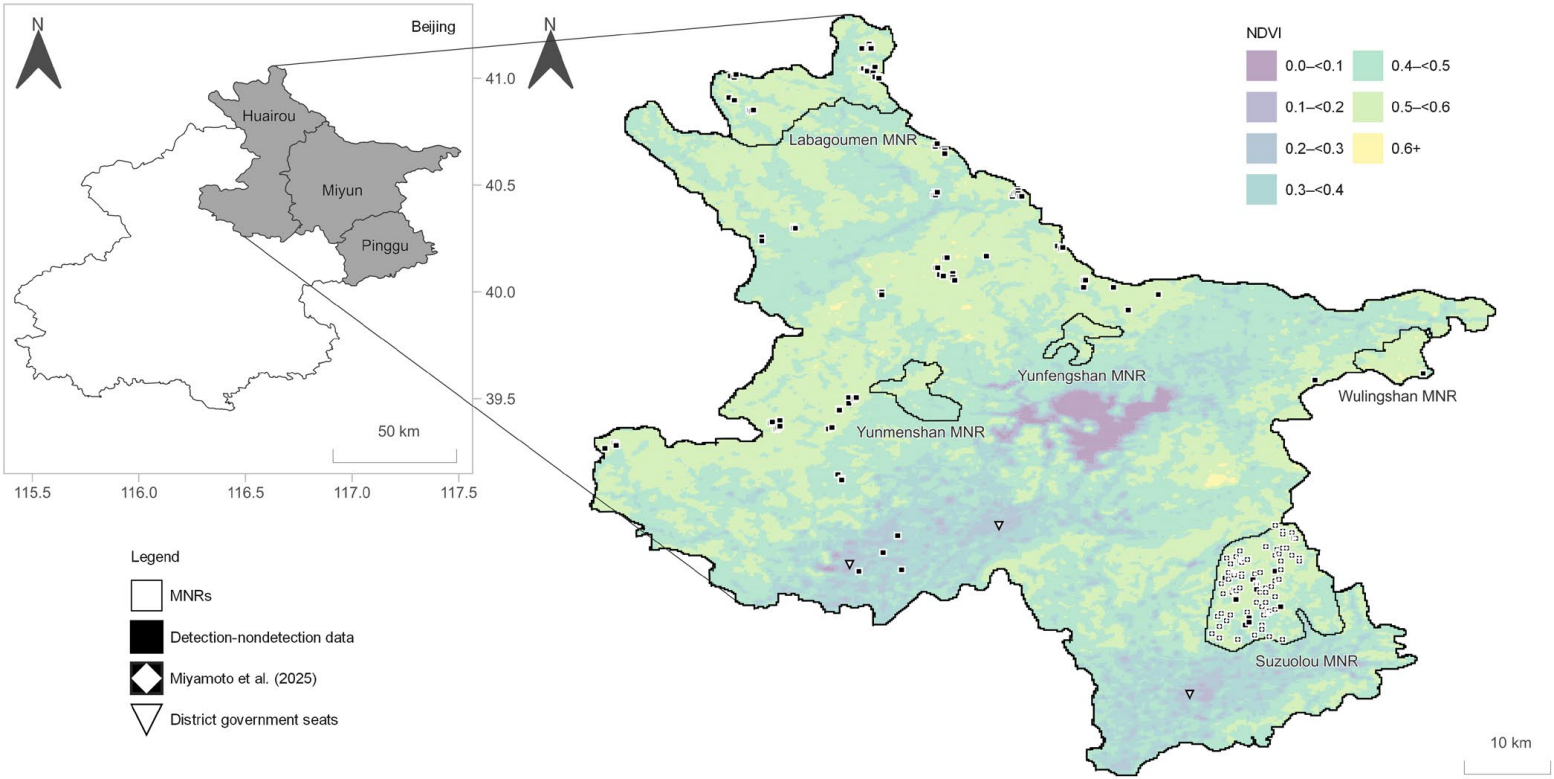
Map of the study area in northeast Beijing, China. The vertical and horizontal axes represent latitude and longitude, respectively. MNR indicates Municipal Nature Reserve, and the names under each polygon indicate the respective reserves. Each grid cell (250 × 250 m) is based on the NDVI raster (Liu et al. 2023), and black grid cells indicate the presence of one or more trail cameras (slightly enlarged for visibility). Of these, 67 grid cells from previous research (Miyamoto et al. 2025) are represented by white diamonds within black grid cells. Data sources: District boundaries from the National Catalogue Service for Geographic Information (www.webmap.cn, accessed on 8 May 2024) and nature reserve polygons from Liang (2021).

The study area comprises three districts, Huairou, Miyun and Pinggu, located in northeast Beijing, China (Figure 1). Beijing has a temperate continental monsoon climate, with a mean annual temperature of 11.5°C and an average annual precipitation of 630 mm. These districts are part of the ecological conservation area designated by Beijing Municipality and provide watershed protection and extensive forest cover. District government seats are located in their southern parts (Figure 1).

### 2.2 Source of Detection–Nondetection Data

We collected detection–nondetection data on raccoon dogs for a 21‐month survey (December 2022–August 2024). Specifically, we used 193 trail cameras: BG636‐48M (Boly Media Communications, China), L710 (YIANWS R&D CENTER, China), and the E3H‐C and E1‐C (Shenzhen Ereagle Technology Co. Ltd., China). To define spatial sampling units and standardise the spatial scale of analysis, we overlaid the study area with 250 × 250 m grid cells, which corresponded to the coarsest spatial resolution of the environmental raster variables. Accordingly, we generated 116,398 grid cells across the study area and sampled detection–nondetection data from 170 of them (Figure 1). Of these, 67 grid cells with detection–nondetection data were obtained from previous research conducted in Pinggu district, Beijing (Miyamoto et al. 2025).

### 2.3 Environmental Variables

We selected 10 environmental variables based on previous raccoon dog studies (Diao et al. 2022; Duscher and Nopp‐Mayr 2017; Miyamoto et al. 2025; Watanabe et al. 2021): naturally regenerating forest, planted forest, grassland, major road density, minor road density, Normalised Difference Vegetation Index (NDVI), elevation, Topographic Position Index (TPI), slope and human population density (Table 1). All selected variables were required to be associated with at least 10% of grid cells with detection–nondetection data to ensure sufficient data support for model estimation. Naturally regenerating forest and planted forest (Bourgoin et al. 2024), and grassland (Zanaga et al. 2022) were available as 10 × 10 m raster data. For each grid cell, we calculated the proportion of raster cells corresponding to each land‐cover type. Based on OpenStreetMap road classifications, roads were grouped into major (motorway, trunk, primary, secondary and tertiary) and minor (residential, unclassified, living_street, service, pedestrian, cycleway, footway, steps, unknown) categories (OpenStreetMap contributors, accessed on 4 April 2025). Major roads are primarily used by motor vehicles, whereas minor roads have other primary uses. Path and track features were excluded from the analysis because they differ from other roads, are often unpaved, and are primarily located in natural areas. We used NDVI as the 2020 annual mean with a 250 m spatial resolution (Liu et al. 2023), and grid cells were derived from this raster. Elevation, TPI and slope were derived from a digital elevation model (DEM; USGS 2018). TPI was calculated within a 50 m radius of each grid cell's centroid (Watanabe et al. 2021). Mean values of these topographic variables were calculated for each grid cell. The human population density was aggregated within each grid cell (Bondarenko et al. 2025).

**TABLE 1:** Environmental variables used in Bayesian occupancy models.

Environmental variables	Unit	Period	Source	VIF
Naturally regenerating forest	10 × 10 m	2020	JRC	4.325
Planted forest	10 × 10 m	2020	JRC	3.997
Grassland	10 × 10 m	2021	ESA	1.242
Major road density	km/km^2^	2021	OSM	1.207
Minor road density	km/km^2^	2021	OSM	1.226
NDVI	250 × 250 m	2020	Liu et al. (2023)	2.720
Elevation	30 × 30 m	2018	USGS	2.402
TPI	30 × 30 m	2018	USGS	1.035
Slope	30 × 30 m	2018	USGS	3.410
Human population density	100 × 100 m	2021	WorldPop	1.351

*Source:* European Commission, Joint Research Centre (JRC) Global Forest Types Map 2020 (Bourgoin et al. 2024), European Space Agency (ESA) WorldCover 2021 v200 (Zanaga et al. 2022), OpenStreetMap (OSM; OpenStreetMap contributors, accessed on 4 April 2025), U.S. Geological Survey (USGS 2018) and WorldPop (Bondarenko et al. 2025).

### 2.4 Bayesian Occupancy Modelling

We used a Bayesian occupancy modelling framework to estimate both grid occurrence (probability that raccoon dogs occurred at a grid) and detectability (probability of detecting the species during a survey replicate) from detection–nondetection data, incorporating spatially structured random effects. We defined the sampling occasion as a 14‐day survey period starting on 3 December 2022. Survey effort was quantified as the number of camera‐trap days per sampling occasion and included as a covariate in the detection submodel. Seasons were defined as winter (December–February), spring (March–May), summer (June–August) and autumn (September–November). Seasons were treated as year‐specific units (i.e., season‐year combinations), and each season comprised four to seven sampling occasions. Occupancy status was assumed to be constant within each season‐year combination. Detection was coded as 1 for a sampling occasion in which the species was recorded, and as 0 otherwise. Grid cells with fewer than three sampling occasions within a season‐year combination were excluded from the analysis to ensure reliability. Sampling occasions were merged with adjacent occasions within the same season if the survey period was at least 7 days, which is half of the 14‐day sampling interval. When more than one camera trap was installed within the same grid cell, detection records and survey effort were aggregated at the grid‐cell level. As random effects, we included season‐year combinations to account for temporal variation and grid cell ID to account for repeated measurements within grid cells.

We developed models in three steps. First, environmental variables were sequentially removed from the full model, and candidate models were compared using the Watanabe‐Akaike Information Criterion (WAIC) to identify the model with the lowest WAIC. Second, variables with highly uncertain posterior estimates were excluded from the lowest WAIC model. These were identified by wide CIs and unstable effect directions, indicating limited information content and poor interpretability (Bondell and Reich 2012). Finally, among the remaining variables, those whose CIs included zero were excluded from the model, as effects that cannot be clearly distinguished from zero provide limited inferential value (van der Pas et al. 2017). After model selection, we generated response curves to evaluate the direction and strength of each variable's effect using 100 evenly spaced values across each variable's range. Using the final model, we predicted relative occupancy probabilities for unsurveyed grid cells across the study area, and we masked grid cells outside the environmental range represented in the training data to avoid extrapolation.

R 4.5.2 (R Core Team 2025) was used for statistical analyses, and QGIS 3.22.7 was utilised for geospatial analyses. We used the R packages ‘spOccupancy’ for a Bayesian occupancy model (Doser et al. 2022) and ‘car’ to compute VIFs to assess multicollinearity among covariates prior to model construction (Fox and Weisberg 2019). We ran 4 MCMC chains, each consisting of 5000 batches of 20 iterations (100,000 iterations per chain). The first 10,000 iterations of each chain were discarded as burn‐in (package default). Other settings were kept at their default values. Convergence was assessed visually using trace and density plots, and quantitatively using standard diagnostics (Gelman‐Rubin statistic [R‐hat] and effective sample size [ESS]), which are commonly used to evaluate the convergence of simulated chains (Vehtari et al. 2021). To account for spatial autocorrelation among sampling sites, we included a spatially structured random effect in the occupancy component of the model (spatial variance and spatial range).

## 3 Results

### 3.1 Occupancy Modelling Performance

All MCMC chains converged satisfactorily for the full model and all reduced models (Table 2), as indicated by R‐hat values below 1.1 and sufficiently large ESS (> 1000). The spatial variance parameter indicated the presence of residual spatial autocorrelation among sampling sites.

**TABLE 2:** Summary of the final Bayesian occupancy model.

Variable	Type	Mean	SD	2.50%	50%	97.50%	R‐hat	ESS
Intercept	Fixed	0.058	0.626	−1.175	0.050	1.328	1.001	7230
Minor road density	Fixed	−0.315	0.176	−0.696	−0.302	−0.003	1.000	22,174
Human population density	Fixed	−0.759	0.367	−1.589	−0.717	−0.163	1.000	14,114
Season‐year	Random	2.470	2.623	0.428	1.763	8.743	1.000	29,391
Grid cell ID	Random	6.481	3.975	0.370	5.886	15.948	1.003	3216
Intercept	Detection	−0.601	0.248	−1.089	−0.600	−0.115	1.000	248,682
Effort	Detection	0.019	0.016	−0.012	0.019	0.051	1.000	291,631
Spatial variance	Spatial	1.300	1.587	0.186	0.713	6.018	1.017	1463
Spatial range	Spatial	0.007	0.004	0.001	0.007	0.014	1.002	5047

### 3.2 Environmental Variables and Predicted Relative Occupancy Probability

The final model retained minor road density (95% CI: −0.696 to −0.003) and human population density (95% CI: −1.589 to −0.163), both of which had 95% CIs excluding zero (Table 2). The posterior mean effects of minor road density and human population density on occupancy were −0.315 and −0.759, respectively. Detection probability showed a positive trend with survey effort (posterior mean = 0.019), although the 95% CI included zero (95% CI: −0.012 to 0.051). Random effects for season‐year and grid cell ID indicated variation in occupancy across time and space.

Predicted relative occupancy probability decreased from 0.444 at zero minor road density to 0.053 at a density of 14.708, whereas it decreased from 0.456 at zero human population density to 0.004 at a density of 25.903 (Figure 3).

### 3.3 Spatial Prediction of Relative Occupancy Probability

Relative occupancy probabilities were predicted across the study area using the final model, ranging from 0 to 0.513 (Figure 3). To avoid extrapolation, 10,666 grid cells were masked and excluded from the prediction map, corresponding to approximately 9% of the study area.

The entire study area averaged 0.394 ± 0.187 (SD). Within non‐extrapolated areas, spatial predictions indicated generally higher relative occupancy probabilities in the northern part than in the southern part. Mean predicted relative occupancy probabilities within municipal nature reserves (MNRs) ranged from 0.421 to 0.481 (Table 3).

**TABLE 3:** Mean predicted relative occupancy probabilities across municipal nature reserves (MNRs).

Name of MNRs	District	Mean	SD	Grid cells
Labagoumen MNR	Huairou	0.481	0.101	4211
Yunmenshan MNR	Miyun	0.480	0.095	1034
Yunfengshan MNR	Miyun	0.477	0.104	592
Wulingshan MNR	Miyun	0.468	0.114	957
Sizuolou MNR	Pinggu	0.421	0.161	4520

*Note:* The Grid cells column indicates the number of grid cells for each MNR.

Predicted relative occupancy probability was generally lower in areas with higher minor road density and human population density, whereas higher values were observed in less disturbed regions (Figures 3 and 4). Predictions also revealed pronounced spatial heterogeneity across the landscape.

## 4 Discussion

### 4.1 Occupancy Modelling Performance

The satisfactory convergence of all MCMC chains, as indicated by enough observations to achieve R‐hat values below 1.1 (Brooks and Gelman 1998) and an adequate ESS above 1000 (Ogawa et al. 2025), suggests that parameter estimates from the occupancy models were stable and reliable. Vehtari et al. (2021) recommend using samples only when R‐hat < 1.01 as an empirical guideline based on extensive experience with MCMC methods. Although the spatial variance slightly exceeded this threshold in the final model, it was close to the empirical guideline (R‐hat = 1.017). This minor deviation is unlikely to affect the interpretation of occupancy patterns or covariate effects, and at least the minimum acceptable level of accuracy was therefore achieved.

The presence of residual spatial autocorrelation indicates that some spatially structured variation in occupancy remained unexplained by the included covariates. This may reflect the influence of unmeasured environmental factors or spatial processes operating at scales not captured by the model.

### 4.2 Environmental Variables and Predicted Relative Occupancy Probability

**FIGURE 2:**
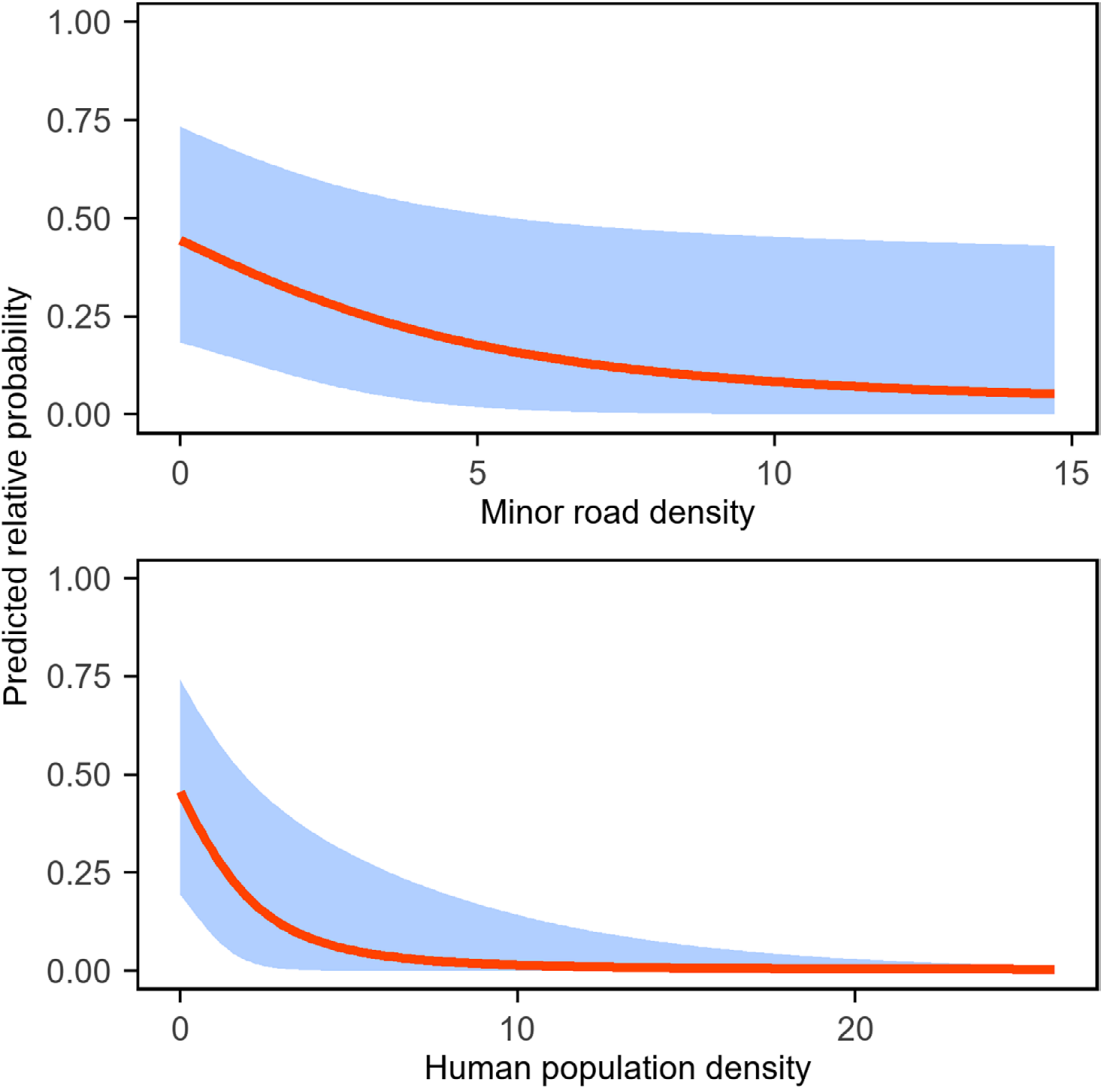
Response curves showing the predicted relative occupancy probability in relation to each environmental variable. Red lines indicate the mean response, and blue shading represents 95% credible intervals.

Our results showed that raccoon dogs had higher predicted relative occupancy probabilities in areas with lower minor road and human population densities, indicating that human‐related disturbance limits their occupancy (Figure 2). Previous studies in Germany have indicated that raccoon dogs avoid anthropogenic structures in lowland rural agricultural landscapes (Drygala et al. 2008; Sutor and Schwarz 2013). However, other studies have found that their distribution is associated with several anthropogenic food resources, including human‐related food and stray cat feeding in Shanghai, China (Diao et al. 2022; Wang et al. 2024; Zhao et al. 2022). Similarly, in Kawasaki, Japan, radio‐telemetry studies have shown that raccoon dogs forage for anthropogenic food resources in residential areas (Yamamoto 1993), and stomach content analyses indicated that these resources accounted for 72.3% of dietary occurrences (Yamamoto and Kinoshita 1994). These findings suggest that raccoon dogs' use of anthropogenic environments is strongly mediated by the availability of anthropogenic food resources. Population growth has been identified as an important driver of municipal solid waste generation, including household food waste (Zambrano‐Monserrate et al. 2021). Given the relatively low human population density in our study area, municipal solid waste was unlikely to provide sufficient anthropogenic food resources for raccoon dogs. As a result, areas with relatively small increases in human population density likely represented increased disturbance without compensatory food benefits, leading to a negative mean effect on occupancy (posterior mean: −0.759; Table 2). For example, human population density (persons km^−2^) in Shanghai (3923) is substantially higher than in our study area, which includes Huairou (207), Miyun (235) and Pinggu (480), based on official statistics (Beijing Municipal Bureau of Statistics 2024; Shanghai Municipal Statistics Bureau 2024).

In rural areas, the landscape structure surrounding roads affects the occurrence of raccoon dog roadkill (Shinoda et al. 2021), and their road‐crossing behaviour appears to be modulated by the surrounding landscape, with more crossings near natural or semi‐natural habitats and fewer crossings in heavily built‐up areas (Choi et al. 2026). Similarly, at broader spatial scales, human structures such as roads and settlements can delay the expansion of alien raccoon dogs in Germany (Sutor and Schwarz 2013). Consistent with these patterns, they occur more frequently in agricultural and wetland areas, closer to water and roads, while showing negative selection coefficients for distance to roads, a pattern interpreted as avoidance of human‐associated disturbance (Melis et al. 2015); raccoon dogs may perceive such disturbance as increased predation risk (Tsunoda et al. 2019). In our study, minor roads were defined as low‐traffic local infrastructure associated with human activities at a fine spatial scale. Accordingly, the negative mean effect of minor roads on occupancy may reflect avoidance of human‐disturbed areas (posterior mean: −0.315; Table 2). However, this result should be interpreted with caution, as the 95% credible interval narrowly approached zero (95% CI: −0.696 to −0.003).

### 4.3 Spatial Prediction of Relative Occupancy Probability

Higher predicted relative occupancy probabilities within nature reserves suggest that these areas may be relatively favourable for raccoon dog habitation (Table 3), as indicated by model predictions incorporating minor road density and human population density (Table 2). This pattern may be relatively stable, given that nature reserves are subject to legal protection that limits development and human access under the Regulations of the People's Republic of China on Nature Reserves.

**FIGURE 3:**
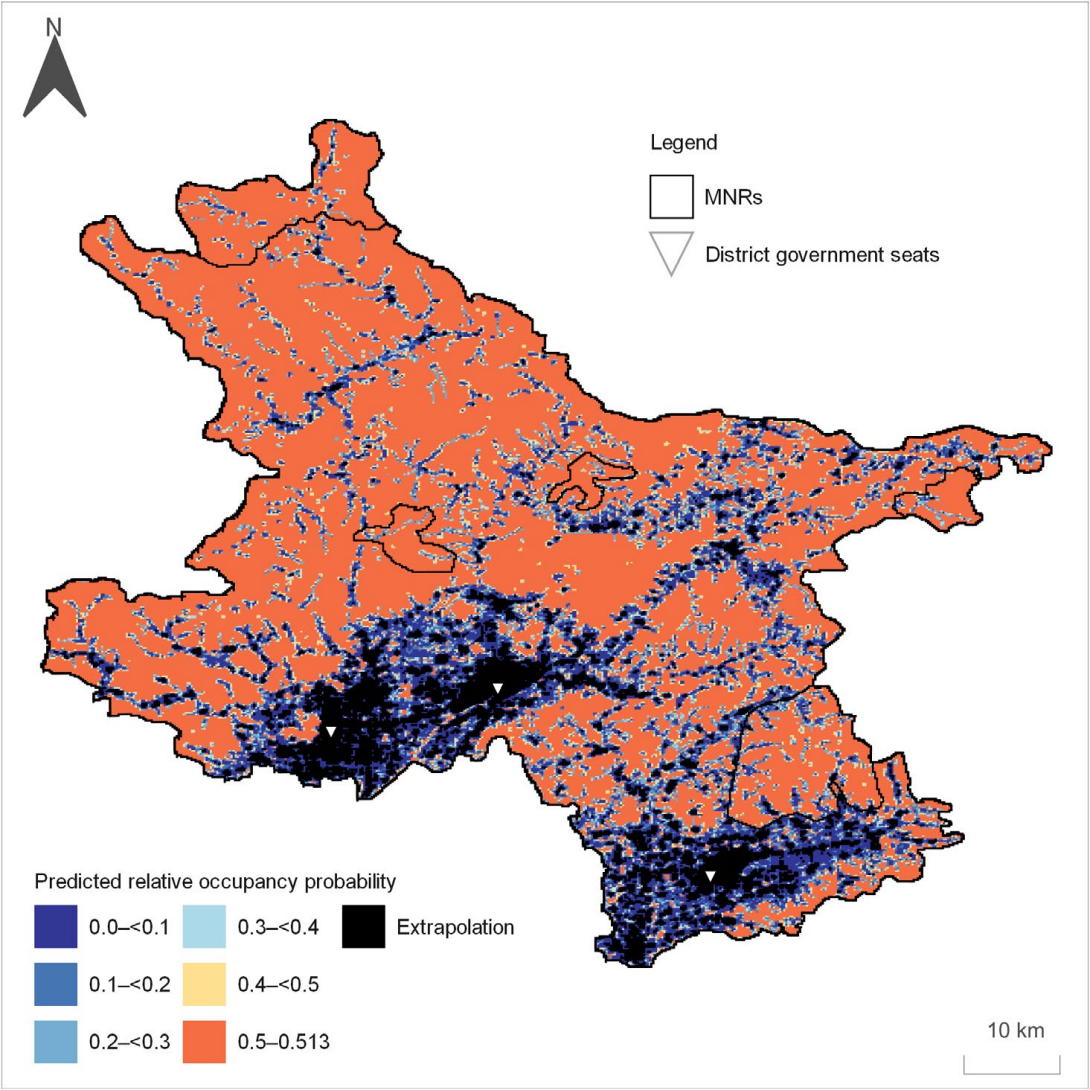
Map of predicted relative occupancy probability for raccoon dogs in northeast Beijing based on Bayesian occupancy models. Black grid cells indicate areas of extrapolation.

**FIGURE 4:**
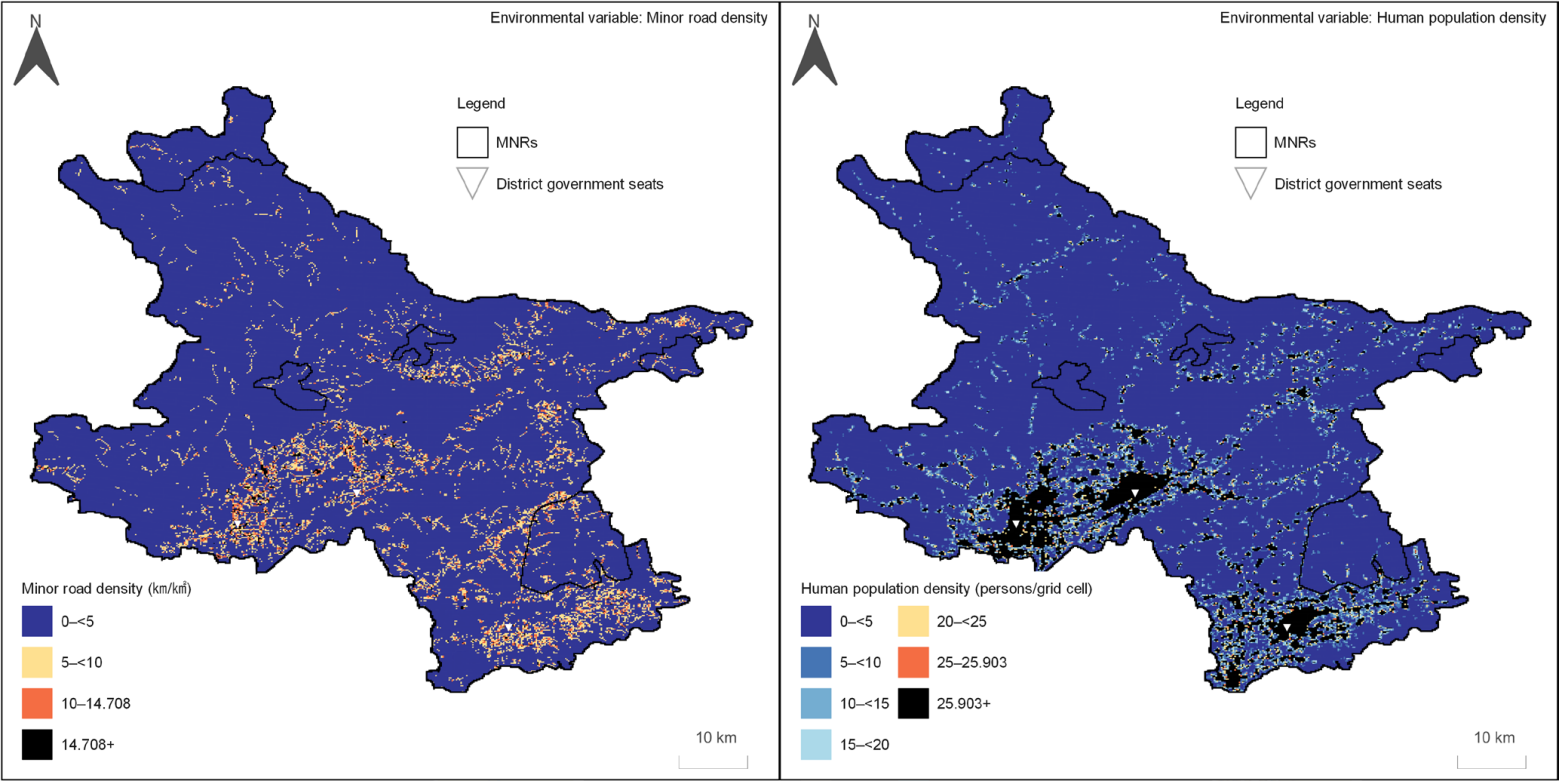
Spatial distribution of environmental variables used in the final model. Black grid cells indicate extrapolated values beyond the range of the final model.

Previous studies showed that raccoon dogs prefer rural and suburban landscapes between forest landscapes and the urban core (Saito and Koike 2013; Saito and Sonoda 2017). Our study area is mainly composed of vegetated rural and forest landscapes (Figure 1), suggesting that it differs from the large‐scale urban landscapes examined in previous studies. However, within non‐extrapolated areas, predicted relative occupancy probabilities tended to decrease in and around district government seats with relatively high human population density (Figures 3 and 4). In contrast, grid cells located along the urban‐forest gradient showed relatively higher predicted occupancy probabilities, reflecting the transitional nature of intermediate landscapes (Figure 1).

In our spatial predictions, extrapolated areas were concentrated in and around district government seats (Figure 3). Our study is based on occupancy modelling using detection–nondetection data obtained from camera traps. Camera traps were installed exclusively at locations where wildlife detections were expected, reflecting practical constraints inherent to camera‐based surveys. Unlike passive presence‐only data, such as museum specimens or opportunistic observation records, detection–nondetection data are typically obtained through active survey designs, for example, systematic transect‐based sampling (Ishihama 2017). However, it is unrealistic to deploy camera traps in human settlement areas. As a result, predictions for these areas relied on model extrapolation beyond the environmental conditions represented in the survey data. Such extrapolation, therefore, likely reflects spatial limitations associated with camera‐trap‐based data collection, rather than an inherent limitation of the occupancy modelling framework itself.

Previous studies have reported that human settlements can act as barriers to raccoon dog expansion (Sutor and Schwarz 2013). Consistent with this, our map showed that human population density and minor road density were concentrated in the southern part of the study area (Figure 4). If these factors limit raccoon dog distribution, the model suggests that raccoon dogs are unlikely to expand southwards towards more densely populated areas or invade central Beijing (Figure 3). Nevertheless, as observed in Shanghai and Kawasaki (Diao et al. 2022; Wang et al. 2024; Yamamoto 1993; Yamamoto and Kinoshita 1994; Zhao et al. 2022), raccoon dogs may be able to expand their distribution through small green spaces, such as parks, if they can utilise anthropogenic resources like garbage. In such cases, human population density may positively influence raccoon dog occurrence until a threshold is reached, consistent with the response curve observed in Shanghai (Diao et al. 2022). Under these conditions, human–wildlife conflicts, such as roadkill, are likely to increase, suggesting that maintaining the current equilibrium may be preferable.

## 5 Conclusion

Based on trail camera data, our findings suggest that raccoon dogs avoid areas disturbed by humans in northeast Beijing. Reflecting these effects, occupancy is lower in the southern parts of the study area, and raccoon dogs may be unlikely to expand southwards into more densely populated areas or invade central Beijing. This study provides the first region‐wide assessment of raccoon dog occupancy in northeast Beijing. A remaining challenge is to expand spatial sampling in human‐dominated landscapes across Beijing by incorporating complementary survey approaches beyond camera trapping, thereby reducing extrapolation in future occupancy modelling.

## Author Contributions

**Keisuke Miyamoto:** conceptualization (lead), data curation (lead), formal analysis (lead), investigation (lead), methodology (lead), validation (lead), visualization (lead), writing – original draft (lead), writing – review and editing (lead). **Xiaofeng Luan:** funding acquisition (lead), project administration (lead).

## Funding

This work was supported by the Beijing Sizuolou Municipal Nature Reserve Supervision Capacity Enhancement Project (Grant No. 2023HKFWBH‐XSH‐02; Principal Investigator: Shaohua Xing, Beijing Forestry University) and the National Key Research and Development Program of China (Grant No. 2022YFF1301405; Principal Investigator: Xiaofeng Luan, Beijing Forestry University).

## Conflicts of Interest

The authors declare no conflicts of interest.

## Supporting information




**Data S1:** ece373192‐sup‐0001‐DataS1.zip.

## Data Availability

All the required data are uploaded as Supporting Information.

## References

[ece373192-bib-0001] An, J. , L. Gai , L. Wang , J. Li , and N. Wang . 2024. “Raccoon Dogs in Beijing Songshan National Nature Reserve [In Chinese].” China Nature 02: 60–63.

[ece373192-bib-0002] Beijing Municipal Bureau of Landscape and Forestry . 2020. “Protected Natural Areas in Beijing.” https://yllhj.beijing.gov.cn/English/Resources/202107/t20210720_2445110.html.

[ece373192-bib-0003] Beijing Municipal Bureau of Statistics . 2024. Beijing Statistical Yearbook. China Statistics Press. https://nj.tjj.beijing.gov.cn/nj/main/2024‐tjnj/zk/indexeh.htm.

[ece373192-bib-0004] Beijing Municipal Ecology and Environment Bureau . 2025. “2024 Report on the State of the Ecology and Environment in Beijing.” https://sthjj.beijing.gov.cn/bjhrb/index/xxgk69/sthjlyzwg/1718880/1718881/1718882/743615203/2025121215323323447.pdf.

[ece373192-bib-0005] Bondarenko, M. , R. Priyatikanto , N. Tejedor‐Garavito , et al. 2025. “Constrained Estimates of 2015–2030 Total Number of People Per Grid Square at a Resolution of 3 Arc (Approximately 100 m at the Equator) R2024B Version v1.” 10.5258/SOTON/WP00803.

[ece373192-bib-0048] Bondell, H. D. , and B. J. Reich . 2012. “Consistent High‐Dimensional Bayesian Variable Selection via Penalized Credible Regions.” Journal of the American Statistical Association 107, no. 500: 1610–1624. 10.1080/01621459.2012.716344. 23482517 PMC3587767

[ece373192-bib-0006] Bourgoin, C. , A. Verhegghen , S. Carboni , et al. 2024. Global Map of Forest Types 2020—Version 0 [Dataset]. European Commission, Joint Research Centre (JRC).

[ece373192-bib-0007] Brooks, S. P. , and A. Gelman . 1998. “General Methods for Monitoring Convergence of Iterative Simulations.” Journal of Computational and Graphical Statistics 7, no. 4: 434–455. 10.1080/10618600.1998.10474787.

[ece373192-bib-0008] Choi, S. , K. Min , S. Cho , et al. 2026. “Movement Patterns of Raccoon Dogs Within Road Networks: How Urbanization Increases Human‐Wildlife Contacts.” Landscape and Urban Planning 268: 105566. 10.1016/J.LANDURBPLAN.2025.105566.

[ece373192-bib-0009] Dayuan, X. , and J. Mingkang . 1994. “A Study on Categorizing Standard of Nature Reserves in China [In Chinese].” China Environmental Science 14, no. 4: 246–251.

[ece373192-bib-0010] Diao, Y. , Q. Q. Zhao , Y. Weng , et al. 2022. “Predicting Current and Future Species Distribution of the Raccoon Dog (*Nyctereutes procyonoides*) in Shanghai, China.” Landscape and Urban Planning 228: 104581. 10.1016/j.landurbplan.2022.104581.

[ece373192-bib-0049] Doser, J. W. , A. O. Finley , M. Kéry , and E. F. Zipkin . 2022. “spOccupancy: An R Package for Single‐Species, Multi‐Species, and Integrated Spatial Occupancy Models.” Methods in Ecology and Evolution 13, no. 8: 1670–1678. 10.1111/2041-210x.13897.

[ece373192-bib-0011] Drygala, F. , N. Stier , H. Zoller , K. Boegelsack , H. M. Mix , and M. Roth . 2008. “Habitat Use of the Raccoon Dog (*Nyctereutes procyonoides*) in North‐Eastern Germany.” Mammalian Biology 73, no. 5: 371–378. 10.1016/j.mambio.2007.09.005.

[ece373192-bib-0012] Duscher, T. , and U. Nopp‐Mayr . 2017. “Species Distribution Modeling for the Invasive Raccoon Dog ( *Nyctereutes procyonoides* ) in Austria and First Range Predictions for Alpine Environments.” Archives of Biological Sciences 69, no. 4: 637–647. 10.2298/ABS161124009D.

[ece373192-bib-0013] Elith, J. , and J. R. Leathwick . 2009. “Species Distribution Models: Ecological Explanation and Prediction Across Space and Time.” Annual Review of Ecology, Evolution, and Systematics 40: 677–697. 10.1146/annurev.ecolsys.110308.120159.

[ece373192-bib-0014] Fan, Y. , J. Yang , H. Zhang , et al. 2020. “Food Composition of Medium Sized Carnivores in Beijing Songshan Nature Reserve [In Chinese].” Acta Theriologica Sinica 37, no. 1: 59–62.

[ece373192-bib-0015] Fithian, W. , and T. Hastie . 2013. “Finite‐Sample Equivalence in Statistical Models for Presence‐Only Data.” Annals of Applied Statistics 7, no. 4: 1917–1939. 10.1214/13-AOAS667. 25493106 PMC4258396

[ece373192-bib-0016] Fourcade, Y. , J. O. Engler , D. Rödder , and J. Secondi . 2014. “Mapping Species Distributions With MAXENT Using a Geographically Biased Sample of Presence Data: A Performance Assessment of Methods for Correcting Sampling Bias.” PLoS One 9, no. 5: 1–13. 10.1371/journal.pone.0097122. PMC4018261 24818607

[ece373192-bib-0017] Fox, J. , and S. Weisberg . 2019. An {R} Companion to Applied Regression. 3rd ed. Sage. https://socialsciences.mcmaster.ca/jfox/Books/Companion/.

[ece373192-bib-0018] Guillera‐Arroita, G. , J. J. Lahoz‐Monfort , J. Elith , et al. 2015. “Is My Species Distribution Model Fit for Purpose? Matching Data and Models to Applications.” Global Ecology and Biogeography 24, no. 3: 276–292. 10.1111/geb.12268.

[ece373192-bib-0019] Ishihama, F. 2017. “Techniques for Presence‐Only Species Distribution Modelling [In Japanese].” Japanese Journal of Conservation Ecology 22: 21–40.

[ece373192-bib-0020] Lau, M. W. N. , J. R. Fellowes , and B. P. L. Chan . 2010. “Carnivores (Mammalia: Carnivora) in South China: A Status Review With Notes on the Commercial Trade.” Mammal Review 40, no. 4: 247–292. 10.1111/j.1365-2907.2010.00163.x.

[ece373192-bib-0021] Liang, X. 2021. “Relationships Between Topographic Heterogeneity and Plant Diversity Based on Protected Area Data: A Case Study of Beijing‐Tianjin‐Hebei Region [In Chinese].” Unpublished doctoral dissertation, Beijing Forestry University. 10.26949/d.cnki.gblyu.2021.001052.

[ece373192-bib-0022] Liu, H. , T. Zhou , and P. Gou . 2023. “NDVI Dataset of China and Average in 361 Cities (250 m, 1990–2020).” 10.3974/geodb.2023.04.06.v1.

[ece373192-bib-0023] MacKenzie, D. I. , J. D. Nichols , G. B. Lachman , S. Droege , A. A. Royle , and C. A. Langtimm . 2002. “Estimating Site Occupancy Rates When Detection Probabilities Are Less Than One.” Ecology 83, no. 8: 2248–2255. 10.1890/0012-9658(2002)083[2248:ESORWD]2.0.CO;2.

[ece373192-bib-0024] Mao, J. , Y. Tian , J. Xie , J. Zhao , L. Ma , and T. Zha . 2021. “Evaluation and Value Estimation of Water Conservation Function of Forest Vegetation of Four Urban Functional Areas in Beijing.” Acta Ecologica Sinica 41, no. 22: 9020–9028. 10.5846/stxb202103290823.

[ece373192-bib-0025] Melis, C. , I. Herfindal , F. Dahl , and P. A. Åhlén . 2015. “Individual and Temporal Variation in Habitat Association of an Alien Carnivore at Its Invasion Front.” PLoS One 10, no. 3: 1–17. 10.1371/journal.pone.0122492. PMC4376685 25815509

[ece373192-bib-0026] Miyamoto, K. , C. Chen , and X. Luan . 2025. “Seasonal Activity Changes in Raccoon Dogs and Influences of Environmental Factors From Autumn to Winter.” Wildlife Biology 6: 1–8. 10.1002/wlb3.01463.

[ece373192-bib-0027] Ogawa, R. , F. Gosselin , K. F. A. Darras , S. Roilo , and A. F. Cord . 2025. “A Classification‐Occupancy Model Based on Automatically Identified Species Data.” Ecology 106, no. 5: e70086. 10.1002/ecy.70086. 40331299 PMC12056683

[ece373192-bib-0028] R Core Team . 2025. “R: A Language and Environment for Statistical Computing.” https://www.r‐project.org/.

[ece373192-bib-0029] Richardson, D. M. , and R. J. Whittaker . 2010. “Conservation Biogeography ‐ Foundations, Concepts and Challenges.” Diversity and Distributions 16, no. 3: 313–320. 10.1111/j.1472-4642.2010.00660.x.

[ece373192-bib-0030] Saito, M. , and F. Koike . 2013. “Distribution of Wild Mammal Assemblages Along an Urban–Rural–Forest Landscape Gradient in Warm‐Temperate East Asia.” PLoS One 8, no. 5: e65464. 10.1371/journal.pone.0065464. 23741495 PMC3669276

[ece373192-bib-0031] Saito, M. , and Y. Sonoda . 2017. “Symptomatic Raccoon Dogs and Sarcoptic Mange Along an Urban Gradient.” EcoHealth 14, no. 2: 318–328. 10.1007/s10393-017-1233-1. 28374159

[ece373192-bib-0032] Shanghai Municipal Statistics Bureau . 2024. Shanghai Statistical Yearbook. China Statistics Press. https://tjj.sh.gov.cn/tjnj/tjnj2024e.htm.

[ece373192-bib-0033] Shinoda, Y. , M. Saeki , M. Takeuchi , and T. Kinoshita . 2021. “Factors Influencing Raccoon Dog Roadkills in the Paddy‐Field Dominated Zone With Landscape Perspective [In Japanese].” Mammalian Science 61, no. 2: 179–187.

[ece373192-bib-0034] Sutor, A. , and S. Schwarz . 2013. “Seasonal Habitat Selection of Raccoon Dogs (*Nyctereutes procyonoides*) in Southern Brandenburg, Germany.” Folia Zoologica 62, no. 3: 235–243. 10.25225/fozo.v62.i3.a10.2013.

[ece373192-bib-0035] Tsunoda, M. , Y. Kaneko , T. Sako , et al. 2019. “Human Disturbance Affects Latrine‐Use Patterns of Raccoon Dogs.” Journal of Wildlife Management 83, no. 3: 728–736. 10.1002/jwmg.21610.

[ece373192-bib-0036] U.S. Geological Survey . 2018. “USGS EROS Archive ‐ Digital Elevation—Shuttle Radar Topography Mission (SRTM) 1 Arc‐Second Global.” 10.5066/F7PR7TFT.

[ece373192-bib-0037] van der Pas, S. , B. Szabó , and A. van der Vaart . 2017. “Uncertainty Quantification for the Horseshoe.” Bayesian Analysis 12, no. 4: 1221–1274. 10.1214/17-BA1065.

[ece373192-bib-0050] Vehtari, A. , A. Gelman , D. Simpson , B. Carpenter , and P.‐C. Bürkner . 2021. “Rank‐Normalization, Folding, and Localization: An Improved Rˆ for Assessing Convergence of MCMC (With Discussion).” Bayesian Analysis 16, no. 2: 667–718. 10.1214/20-ba1221.

[ece373192-bib-0039] Wang, L. , J. Zhao , and X. Guo . 2015. “Thoughts on Establishing the Monitoring System of Biological Diversity in Nature Reserve [In Chinese].” Journal of Shandong Forestry Science and Technology 45, no. 6: 97–100.

[ece373192-bib-0040] Wang, Y. , Q. Zhao , L. Tang , et al. 2024. “Behavioral Plasticity of Raccoon Dogs (*Nyctereutes procyonoides*) Provides New Insights for Urban Wildlife Management in Metropolis Shanghai, China.” Environmental Research Letters 19, no. 10: 104063. 10.1088/1748-9326/ad7309.

[ece373192-bib-0041] Watanabe, K. , N. Kumagai , and M. Saito . 2021. “Latrine Site Selection of Raccoon Dogs in a Hilly Area in North‐Eastern Japan.” Zoology and Ecology 31, no. 2: 79–85. 10.35513/21658005.2021.2.2.

[ece373192-bib-0042] Yamamoto, Y. 1993. “Home Range and Diel Activity Pattern of the Raccoon Dog, *Nyctereutes procyonoides viverrinus* in Kawasaki [In Japanese].” Bulletin of the Kawasaki Municipal Science Museum for Youth 4: 7–12.

[ece373192-bib-0043] Yamamoto, Y. , and A. Kinoshita . 1994. “Food Composition of the Raccoon Dog *Nyctereutes procyonoides viverrinus* in Kawasaki [In Japanese].” Bulletin of the Kawasaki Municipal Science Museum for Youth 5: 29–34.

[ece373192-bib-0044] Zambrano‐Monserrate, M. A. , M. A. Ruano , and V. Ormeño‐Candelario . 2021. “Determinants of Municipal Solid Waste: A Global Analysis by Countries' Income Level.” Environmental Science and Pollution Research 28, no. 44: 62421–62430. 10.1007/s11356-021-15167-9. 34196869

[ece373192-bib-0045] Zanaga, D. , R. Van De Kerchove , D. Daems , et al. 2022. “ESA WorldCover 10 m 2021 v200.” Zenodo. 10.5281/zenodo.7254221.

[ece373192-bib-0046] Zhang, Y. 2018. “Study of Biodiversity Monitoring System on the Forest Ecosystem Nature Reserves.” Forest Resources Management 3: 29–34. 10.13466/j.cnki.lyzygl.2018.03.006.

[ece373192-bib-0047] Zhao, Q. , Y. Diao , Y. Weng , et al. 2022. “Predicting Future Distributions and Dispersal Pathways for Precautionary Management of Human‐Raccoon Dog Conflicts in Metropolitan Landscapes.” Environmental Research Letters 17, no. 10: 104036. 10.1088/1748-9326/ac9491.

